# Multifunctional Composite Hydrogels for Bacterial Capture, Growth/Elimination, and Sensing Applications

**DOI:** 10.1021/acsami.2c08582

**Published:** 2022-10-12

**Authors:** Andrea Dsouza, Chrystala Constantinidou, Theodoros N. Arvanitis, David M. Haddleton, Jérôme Charmet, Rachel A. Hand

**Affiliations:** † Warwick Manufacturing Group, The University of Warwick, Coventry, United Kingdom CV4 7AL; ‡ Warwick Medical School, The University of Warwick, Coventry, United Kingdom CV4 7AL; § Department of Chemistry, The University of Warwick, Coventry, United Kingdom CV4 7AL; ∥ School of Engineering—HE-Arc Ingénierie, HES-SO University of Applied Sciences Western Switzerland, 2000 Neuchâtel, Switzerland; ⊥ Institute of Digital Healthcare, Warwick Manufacturing Group, The University of Warwick, Coventry, United Kingdom CV4 7AL

**Keywords:** multifunctional hydrogels, bacterial capture elements, bacterial adhesion, bioactive elements, hydrogel-embedded carriers, active hydrogels, functionalized hydrogels, interfaced sensors, therapeutic hydrogels

## Abstract

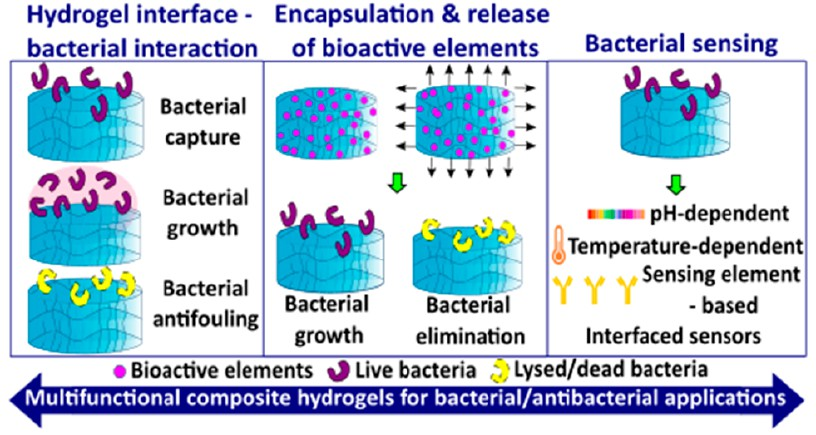

Hydrogels are cross-linked networks of hydrophilic polymer chains with a three-dimensional structure. Owing to their unique features, the application of hydrogels for bacterial/antibacterial studies and bacterial infection management has grown in importance in recent years. This trend is likely to continue due to the rise in bacterial infections and antimicrobial resistance. By exploiting their physicochemical characteristics and inherent nature, hydrogels have been developed to achieve bacterial capture and detection, bacterial growth or elimination, antibiotic delivery, or bacterial sensing. Traditionally, the development of hydrogels for bacterial/antibacterial studies has focused on achieving a single function such as antibiotic delivery, antibacterial activity, bacterial growth, or bacterial detection. However, recent studies demonstrate the fabrication of multifunctional hydrogels, where a single hydrogel is capable of performing more than one bacterial/antibacterial function, or composite hydrogels consisting of a number of single functionalized hydrogels, which exhibit bacterial/antibacterial function synergistically. In this review, we first highlight the hydrogel features critical for bacterial studies and infection management. Then, we specifically address unique hydrogel properties, their surface/network functionalization, and their mode of action for bacterial capture, adhesion/growth, antibacterial activity, and bacterial sensing, respectively. Finally, we provide insights into different strategies for developing multifunctional hydrogels and how such systems can help tackle, manage, and understand bacterial infections and antimicrobial resistance. We also note that the strategies highlighted in this review can be adapted to other cell types and are therefore likely to find applications beyond the field of microbiology.

## 1 Introduction

Hydrogels are a network of hydrophilic cross-linked polymer chains that can possess above 90% water retaining capacity and a distinct three-dimensional structure. One of the main advantages of hydrogels is their versatility provided by unique tunable properties such as porosity, swelling, mechanical strength, stiffness, viscoelasticity, permeability, biocompatibility, biodegradability, and microenvironmental sensing. Previous reviews^1−6^ provide a comprehensive outline of hydrogel chemistry, synthesis, structure, and function. In this review we concentrate on microbiology applications. Since their first description of hydrogels by Wichterle and Lím in 1960,^7^ hydrogels have been extensively used in a wide range of biomedical applications such as drug delivery,^8−11^ tissue engineering,^12−15^ wound dressings,^16−20^ and biosensing.^21−24^ Over the years, with the surge in bacterial infections associated with rapidly evolving antimicrobial resistance, the diagnostics and management of bacteria-associated infectious diseases has become challenging. Hydrogels, due to their versatility and enabling features, provide a favorable platform for a wide range of bacterial/antibacterial applications. In this context, hydrogels have been developed for antibacterial activity, for targeted antibiotic delivery, and in recent years, for the detection of specific causative bacterial species, typically by exploiting/altering their inherent properties (Table 1).

**Table 1:** Enabling Features of Hydrogels for Bacterial/Antibacterial Applications as Identified in This Review

	features		
functions	chemical modifications	(inherent) physicochemical and structural features	encapsulation/release
selective bacterial capture (section 2)	surface	charges, functionalization	bacterial capture elements
bacterial adhesion, growth, and antifouling (section 3)	network	stiffness, thickness, porosity, hydrophilicity/hydrophobicity, charges	growth promoting/inhibiting agents
antibiotic delivery (section 4)	network	porosity, hydrophilicity	antibiotics, carriers
antibacterial activity and treatment of infections (section 4)	surface, network	polymer nature, charges	bactericidal agents
bacterial sensing (active/passive) (section 5)	surface, network	structure reversibility, glass transition temperature, response to external stimuli	pH indicators, bacterial viability dyes, bacterial recognition elements

**Figure 1:**
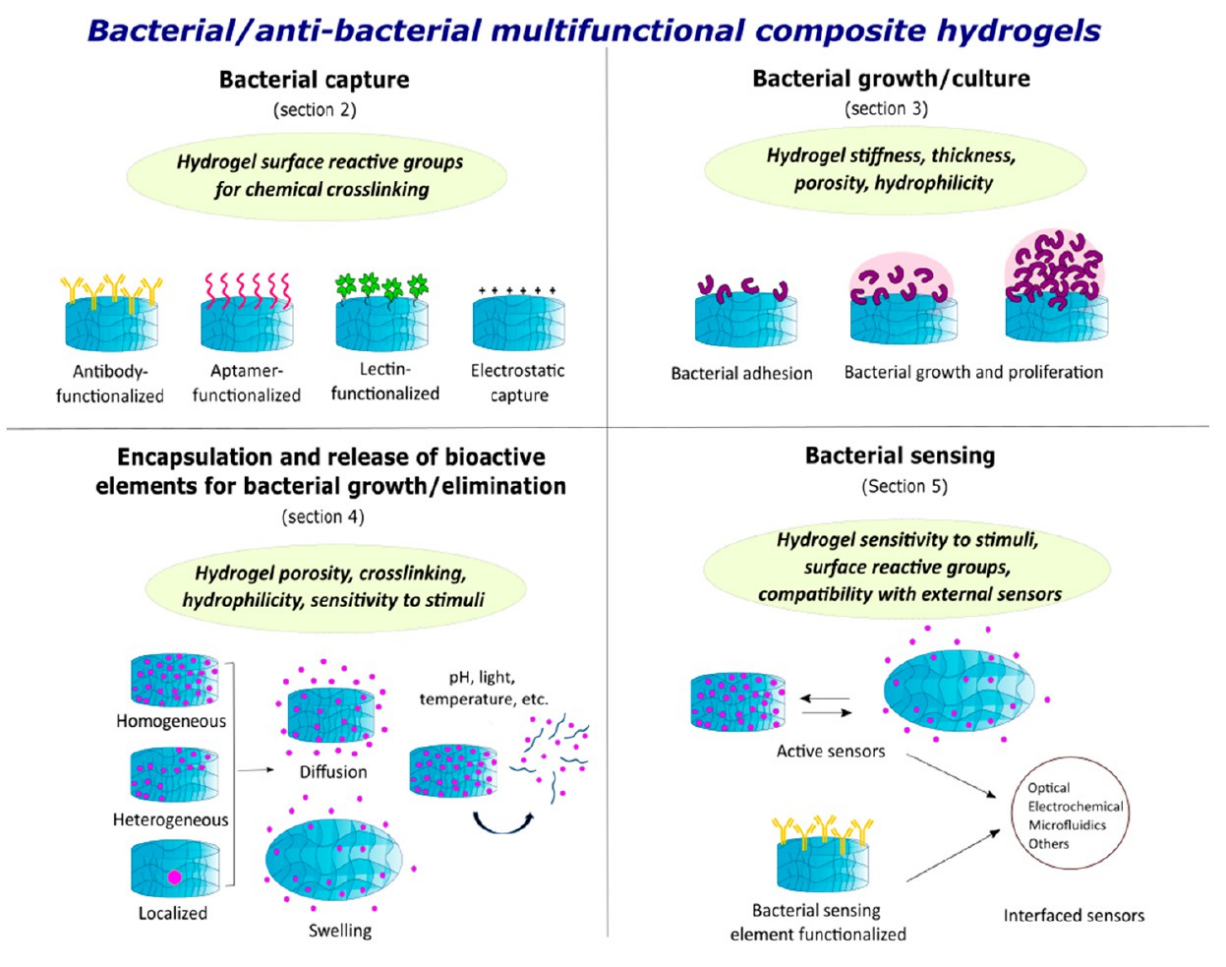
Hydrogel properties and mechanisms/functionalization for developing bacterial/antibacterial hydrogel platforms. Hydrogel properties and their respective bacterial/antibacterial applications are discussed in the text, in the corresponding sections indicated.

Often, the functions described in Table 1 need to be used in combination. For instance, biosensing, enrichment, or elimination steps are often preceded by the selective capture of the target bacteria. One strategy for the development of such a multifunctional system typically relies on sequentially combining multiple single functionalized hydrogels that perform synergistically. This is the case where the surface of the first hydrogel is modified for bacterial capture while the second hydrogel, in close proximity to the first, releases the encapsulated antibacterial elements upon bacterial capture.^25^ Other examples, described later, rely on endowing a single hydrogel with multiple functions. The possibility to combine a range of functions in so-called multifunctional hydrogels paves the way for the development of smart, yet robust and simple solutions for biomedical and diagnostic applications. Figure 1 summarizes the enabling properties of hydrogels essential for the development of bacterial/antibacterial hydrogel platforms.

This review aims to present the recent advances in the development and application of hydrogels designed to perform multiple functions for bacterial/antibacterial studies and sensing. We particularly concentrate on hydrogel properties and strategies for enabling selective bacterial capture, bacterial growth, efficient encapsulation and release of bioactive elements, and bacterial sensing based on active and passive hydrogel systems. Following this, the emerging field of multifunctional composite hydrogels and the hybrid approaches currently pursued for their development are reviewed. It is noted that even though this review concentrates on bacterial/antibacterial applications, many processes and concepts can be applied to other biomedical and diagnostic disciplines.

## 2 Selective Bacterial Capture

Bacterial capture and enrichment are significant especially in diagnostic applications, to specifically identify pathogenic bacteria and perform downstream testing. Typically, bacteria reside in a pool of different bacterial species, cell types, nucleic acids, and molecules in biofluids (blood, urine, sputum, and saliva). Thus, a wide range of bacterial recognition and capture systems have been developed to isolate the bacteria of interest from a complex mixture.^26−29^ However, the current systems are often laborious and expensive due to extensive processing.

A significant advantage of hydrogels is that they offer a great potential for bacterial recognition and capture due to the abundance of chemically reactive groups on their surface/network. Bacterial capture elements (BCEs) can be covalently immobilized on a hydrogel surface or network via chemical cross-linkers.^25,30−32^ However, a hydrogel surface immobilization of BCEs is often preferable as it enables direct interaction between bacteria and its capture element compared to network immobilization. In addition to BCEs, the intrinsic hydrogel surface charges have been exploited to electrostatically attract and enrich bacteria.^33,34^ In this section, we highlight the most commonly used BCEs and strategies for their immobilization on hydrogel surfaces. Finally, the use of hydrogel surface charges for bacterial recognition and capture is described.

### 2.1 Bacterial Capture Elements

Bacterial capture is facilitated by recognition molecules, known as BCEs, that typically interact and bind with bacterial surface markers. Such elements can be chemically coupled on hydrogel surfaces. Immobilization of BCEs on hydrogel surfaces enables specific bacterial capture. In addition, hydrogels offer large surface areas with multiple tethering points to immobilize BCEs. Typically, antibodies, aptamers, and lectins have been used as BCEs for bacterial immobilization on hydrogel surfaces.

Antibodies (monoclonal, polyclonal, and recombinant) are some of the most widely used BCEs for hydrogel functionalization.^32,35−37^ They are immune-based glycoprotein molecules that bind noncovalently to antigens present on the bacterial cell surface/flagella. While antibodies have been successful BCEs due to their accurate and sensitive bacterial detection features, they are often expensive as their manufacturing process involves the use of animals. In addition, the process is time-consuming and often produces batch-to-batch structural variants. Moreover, antibodies are susceptible to relatively rapid degradation and thus exhibit limited shelf life and thermal stability.

An interesting alternative is aptamers, which are short single-stranded synthetic oligonucleotides or peptide sequences that noncovalently interact with bacterial cell surface antigens. Aptamers are synthesized and selected from a library of diverse nucleotide/peptide sequences by a procedure known as systematic evolution of ligands by exponential enrichment (SELEX). Once their sequence is determined, these molecules are easy to synthesize with consistent batch-to-batch production, long shelf life, good stability, and cost-effectiveness.^38^ The development of aptamer-functionalized hydrogels is recently emerging, and studies indicate successful bacterial recognition and capture as highlighted in section 2.2. While aptamers are seemingly well-suited BCEs due to their aforementioned characteristics, they often need stringent validation during the SELEX procedure typically in nucleotide-based aptamers, due to their negative charges hindering their interaction with negatively charged bacteria.^39,40^

Lectins are another type of BCE that has been widely used for bacterial recognition and capture.^41−44^ They are naturally occurring proteins, found in bacterial cell walls and plants. Several studies demonstrate efficient bacterial capture platforms based on lectin–hydrogel surface functionalization. Lectins can be used (1) as a BCE, by taking advantage of their inherent carbohydrate binding domain to interact with glycoproteins and sugar residues present in the bacterial cell wall,^25,45^ or (2) as target molecules.^46^ In the former, lectins are immobilized on hydrogels to capture bacteria. In the latter, hydrogels are functionalized with carbohydrate molecules to recognize lectins present in the bacterial cell wall. For example, a supramolecular hydrogel (discussed in section 6.2.2) functionalized with galactose residues was developed for the binding and inhibition of *Pseudomonas aeruginosa* (*P. aeruginosa*).^46^ An important advantage of lectins is their ability to capture multiple bacteria due to their multimeric structures. Lectins share many advantages with antibodies and aptamers; however, they often lack specificity toward capture of individual bacterial species/strain due to a wide distribution of glycoproteins/sugars commonly occurring in most bacterial cell walls.

Other elements with bacterial capture abilities include bacteriophages,^47−49^ peptides,^50−52^ chemical compounds,^53,54^ positively charged magnetic nanoparticles, and aptamer/lectin-coated magnetic beads.^55,56^ Such BCEs can also potentially be functionalized on hydrogel surfaces for bacterial capture.

### 2.2 Hydrogel Functionalization with BCEs

Functionalization of hydrogels with BCEs typically involves surface functionalization, performed postsynthesis. Hydrogel surfaces can harbor various chemically reactive groups such as amine (−NH_3_), aldehyde (−CHO), carboxyl (−CHO), and thiol (−SH) which provide a suitable interface to immobilize BCEs. In addition, protein-based hydrogels such as albumin, gelatin, and silk offer many reactive groups, mostly amines that are inherent to the peptide backbone, suited for immobilizing BCEs. Hydrogel surface modification via chemical cross-linking is essential as many reactive groups of hydrogels are inactive, requiring activation for immobilizing BCEs. Typically, BCEs are immobilized on a hydrogel surface via the introduction of chemical cross-linkers that activate hydrogel reactive groups by amide bonding/thiol interactions. The activated hydrogels subsequently react with BCEs typically via amine–amine interactions enabling immobilization of BCEs on hydrogels.

**Figure 2:**
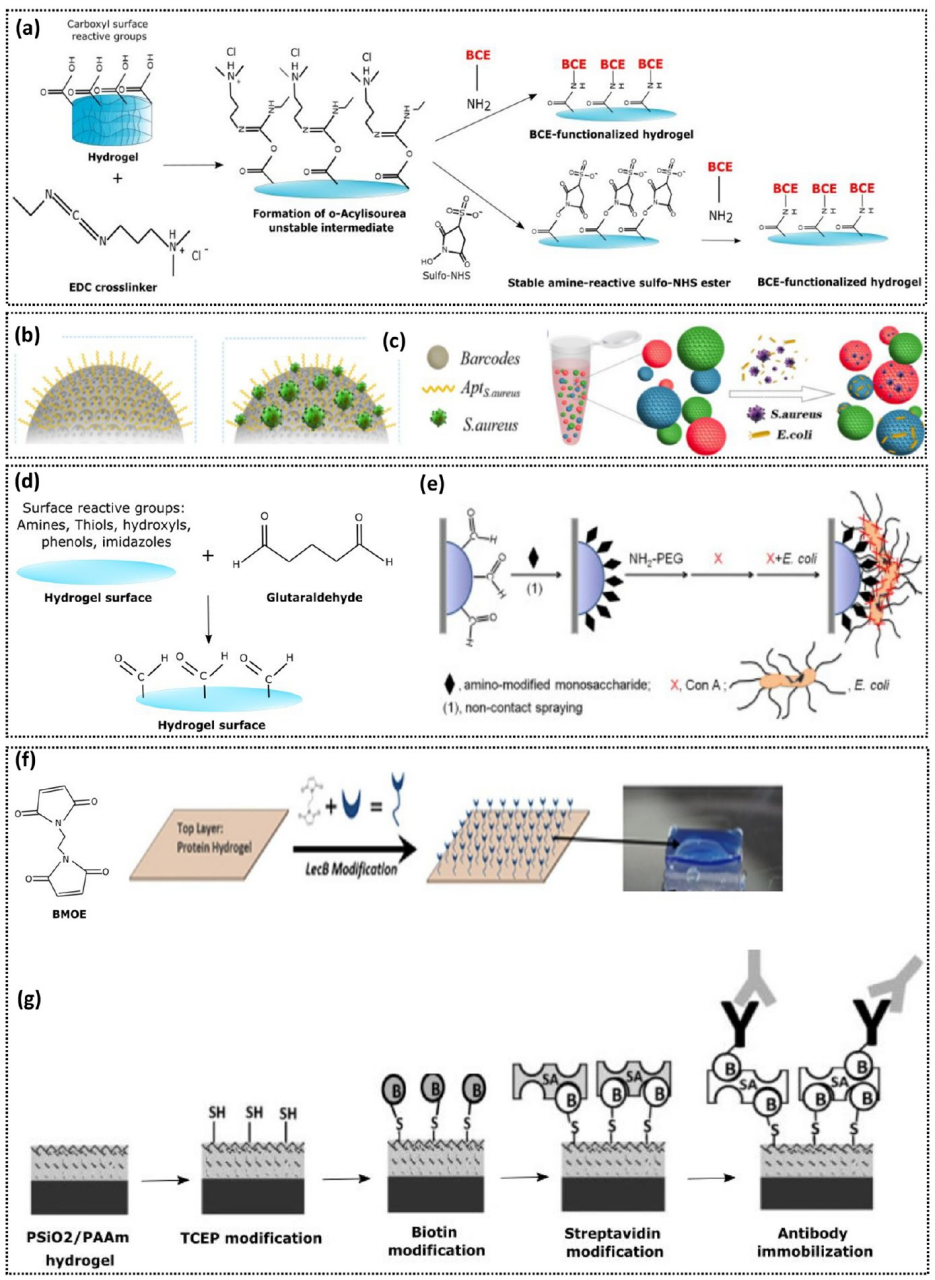
Surface functionalization strategies to attach BCEs to hydrogels. (a) Schematic illustrating BCE immobilization via EDC-NHS chemistry. EDC reacts with the carboxyl groups of the hydrogel, forming an unstable intermediate for interaction with BCEs. Addition of NHS improves the cross-linking efficiency by the formation of stable intermediates for interaction with BCEs. (b, c) Schematic representation of aptamer decorated PEG-hydrogel barcodes for specific bacterial capture: *E. coli* and *S. aureus* from complex biofluid. (a–c) Reprinted with permission from ref (30). Copyright 2018 Elsevier Ltd. (d) GA activation of hydrogel surfaces to produce carbonyl interface for BCE immobilization. (e) From left to right: GA activated hydrogel for tethering monosaccharides, followed by NH_2_-PEG blocking to occupy unreacted sites. Con A immobilizes on hydrogels via monosaccharides, facilitating bacterial capture. Reprinted with permission from ref (45). Copyright 2014 Elsevier Ltd. (f) BMOE modification of protein hydrogel to immobilize lectins for bacterial capture. From ref (25). CC BY 4.0. (g) Thiol modification of PSiO_2_/PAAm hydrogels for biotin/streptavidin conjugation on hydrogel surface to immobilize antibodies for bacterial capture. Reprinted with permission from ref (35). Copyright from 2010 John Wiley & Sons.

One of the most widely used cross-linkers for biomedical applications is 1-ethyl-3-(3-dimethylaminopropyl) carbodiimide hydrochloride (EDC) due to its affordability, noncytotoxicity, and biocompatibility features.^57−60^ It is a hydrophilic carbodiimide cross-linker that reacts with the carboxyl groups of hydrogels and forms an unstable *O*-acylisourea intermediate. In the presence of BCEs, the amine groups of BCEs interact with the unstable intermediate of the EDC-activated hydrogel via amide bonding, causing hydrolysis of the intermediate (Figure 2a). Due to their weak stability, *O*-acylisourea intermediates are easily hydrolyzed in polar solvents and are thus rapidly inactivated. Classed as a zero-length cross-linker, EDC does not introduce any additional atoms between the conjugating moieties. To improve the efficiency of the EDC cross-linking reaction, typically, sulfo-*N*-hydroxysulfosuccinimide (sulfo-NHS) is added onto EDC-activated hydrogels producing a stable ester intermediate which does not hydrolyze rapidly. In the presence of BCEs, the ester intermediate is hydrolyzed due to the covalent attachment of BCEs with the carboxyl groups of EDC-NHS activated hydrogels (Figure 2a). Addition of sulfo-NHS enhances the chemical cross-linking reaction, improves the cross-linking yield, and prevents the formation of chemical byproducts.^61^ Studies indicate successful immobilization of BCEs on hydrogels via EDC-NHS chemistry for bacterial capture and downstream applications.^30−32^ However, sulfo-NHS is known to exhibit cytotoxicity and thus may be incompatible for nonbacterial capture, involving *in vitro* and *in situ* applications^62^ such as targeted antibiotic delivery and wound healing. As an example of EDC-NHS BCE functionalization, poly(ethylene glycol) (PEG) hydrogels immobilized with bacterial species specific aptamers as BCEs were developed for the capture and detection of *Staphylococcus aureus* (*S. aureus*) and *Escherichia coli* (*E. coli*) (Figure 2b,c).^30^ The EDC-NHS–aptamer hydrogels were developed as magnetic inverse opal barcodes with characteristic reporter molecules for specific bacterial identification and capture. The hydrogel platform was equipped with additional features, wherein the barcodes were integrated with magnetic nanoparticles to gain control over bacterial capture under the magnetic field. This study indicated the development of an efficient bacterial “capture and sense” hydrogel platform which could potentially be utilized for diagnosing bacterial infections by selective capture and distinction of bacterial species.

Another cross-linker alternative is glutaraldehyde (GA), a bifunctional cross-linker that reacts with the hydrogel surface reactive groups such as amines, thiols, hydroxyls, phenols, and imidazoles to provide a carbonyl interface that can be used to immobilize BCEs (Figure 2d). However, GA is cytotoxic and nonbiocompatible as it introduces additional atoms between conjugating moieties which are unsafe for biomedical applications. As an example of GA-BCE functionalization, amino-modified monosaccharides, namely 4-aminophenyl α-d-mannopyranoside (Man-α), 4-aminophenyl β-d-galactopyranoside (Gal-β), and 4-aminophenyl β-d-glucopyranoside (Glc-β), were immobilized on polyacrylamide (PAAm) hydrogel via GA hydrogel surface modification to tether the lectin concanavalin A (con A) BCE.^45^ The BCEs interact with the GA-immobilized monosaccharides via amine–amine interactions, resulting in their attachment on the hydrogels (Figure 2e). This study demonstrated the fabrication of a bacterial capture microreactor that enabled high *E. coli* capture efficiency due to the multivalent binding nature of concanavalin A lectins. GA has also been utilized for immobilizing antibodies on hydrogel surfaces. For example, antibodies were immobilized on graphene–chitosan hydrogel nanocomposites for capture and detection of marine sulfate reducing bacteria.^37^

Studies also indicate the use of reducing agents to activate hydrogels that are rich in redox reactive groups such as sulfides and sulfhydryls for BCE immobilization. For instance, a porous silicon dioxide (PSiO_2_) disulfide linked hydrogel was treated with tris(2-carboxyethyl) phosphine (TCEP), which reduces disulfide bonds and activates thiol groups in the hydrogel.^35^ The thiol activated hydrogel enables conjugation of biotin–streptavidin on the hydrogel, thereby enabling antibody interaction and immobilization as shown in Figure 2g. Another study demonstrated immobilization of lectin B BCEs on bovine serum albumin hydrogels, a protein-based hydrogel via bismaleimidoethane (BMOE) cross-linker (Figure 2f).^25^ The cross-linker enables immobilization of lectins on hydrogels by conjugating with the sulfhydryl groups of the protein hydrogel.

In cases where BCEs are not an option, or when less specific (i.e., broader) bacterial capture is required, the surface charge properties of bacteria and hydrogels can be exploited for capture and enrichment. Bacterial cells are negatively charged due to the presence of carboxyl, amine, and teichoic acid groups present in their peptidoglycan layer. Thus, positively charged hydrogels can be utilized/developed to electrostatically interact with bacteria. Studies indicate the use of chitosan-based modified hydrogels for improved charge-based bacterial enrichment by enhancing the intrinsically positive nature of chitosan.^33,34,63^ Such hydrogel platforms have been developed for multifunctional applications for bacterial elimination after capture. An example includes the use of chitosan hydrogels that can electrostatically attract bacteria and then subsequently induce bactericidal effects through a yet to be understood complex sequence of bacterial lysis events.^63^ To improve bacterial capture via electrostatic attraction, chitosan hydrogels were modified with the use of glycidyltrimethylammonium chloride (GTAC) and glycidyl methacrylate (GMA).^34^ The modified chitosan hydrogels electrostatically trapped *S. aureus* and *E. coli* and exhibited antibacterial effects upon capture due to the inherent antibacterial properties of chitosan. Another study demonstrated the development of pNIPAAM poly(*N*-isopropylacrylamide)–AAM (acrylamide)–DMPA (*N*-[3-(dimethylamino)propyl] methacrylamide) hydrogels with molybdenum disulfide (MoS_2_) to electrostatically capture and confine bacteria.^33^ The positively charged MoS_2_ attracts bacteria and exerts an additional antibacterial effect due to its inherent antibacterial nature. While electrostatic bacterial capture and enrichment is a simple and rapid solution, it offers limited potential for the development of robust hydrogel–bacterial capture platforms. Indeed, it does not allow for specific bacterial capture in complex biofluids consisting of more than one bacterial species. In addition, electrostatic adsorption is weak compared to chemical cross-linking strategies due to reversible noncovalent capture which may reduce bacterial capture efficiencies. Finally, electrostatic capture may not be feasible for multifunctional composite hydrogels designed to encapsulate various active elements such as redox indicators and nutrients which also contain charged groups that can potentially interfere with bacterial capture.

## 3 Hydrogels Favoring Bacterial Adhesion/Antifouling

The aqueous microenvironment of hydrogels, their porosity, and the ability to modulate their physicochemical characteristics provide a favorable interface for bacterial interaction and adhesion (explained below). Additionally, these features can potentially be exploited for evaluating bacterial attachment, growth, and biofilm formation. Bacterial adhesion on hydrogel surfaces is a complex process governed by multiple factors^64^ including hydrogel stiffness,^65−69^ porosity,^70,71^ thickness,^65,71,72^ surface roughness,^69,73,74^ hydrophilicity,^75−77^ nutrient medium,^66,78^ and bacterial motility.^67,79^

Typically, bacteria attach on surfaces initially via “reversible adhesion”, where they interact with surfaces through long-range and short-range interactions based on the distance between bacteria and the hydrogel surface.^80−82^ The forces driving bacterial adhesion involve van der Waals forces, electrostatic attraction, Brownian motion, gravitational forces, hydrophobic interactions, hydrogen bonding, ionic and dipole interactions.^80^ The next stage of adhesion involves the firm anchorage of bacteria onto hydrogel surfaces known as “irreversible adhesion” due to the secretion of extrapolymeric substances by bacterial cells that establishes its covalent attachment on the hydrogel surface. Furthermore, interaction of bacterial cellular appendages such as flagella, pili, capsules, and slime constituting polysaccharide adhesins with the hydrogel drives this stage of bacterial adhesion.^80,82^ It is important to note that the process of bacterial adhesion on hydrogels is not universally applicable for all bacterial species/strains since each bacterium possesses unique cellular characteristics. However, it is possible to manipulate hydrogel features to enable both bacterial growth and proliferation, and bacterial antifouling for example, to prevent bacterial adhesion on urinary catheters, dental root fillings, contact lenses, and dermal fillers.

**Figure 3:**
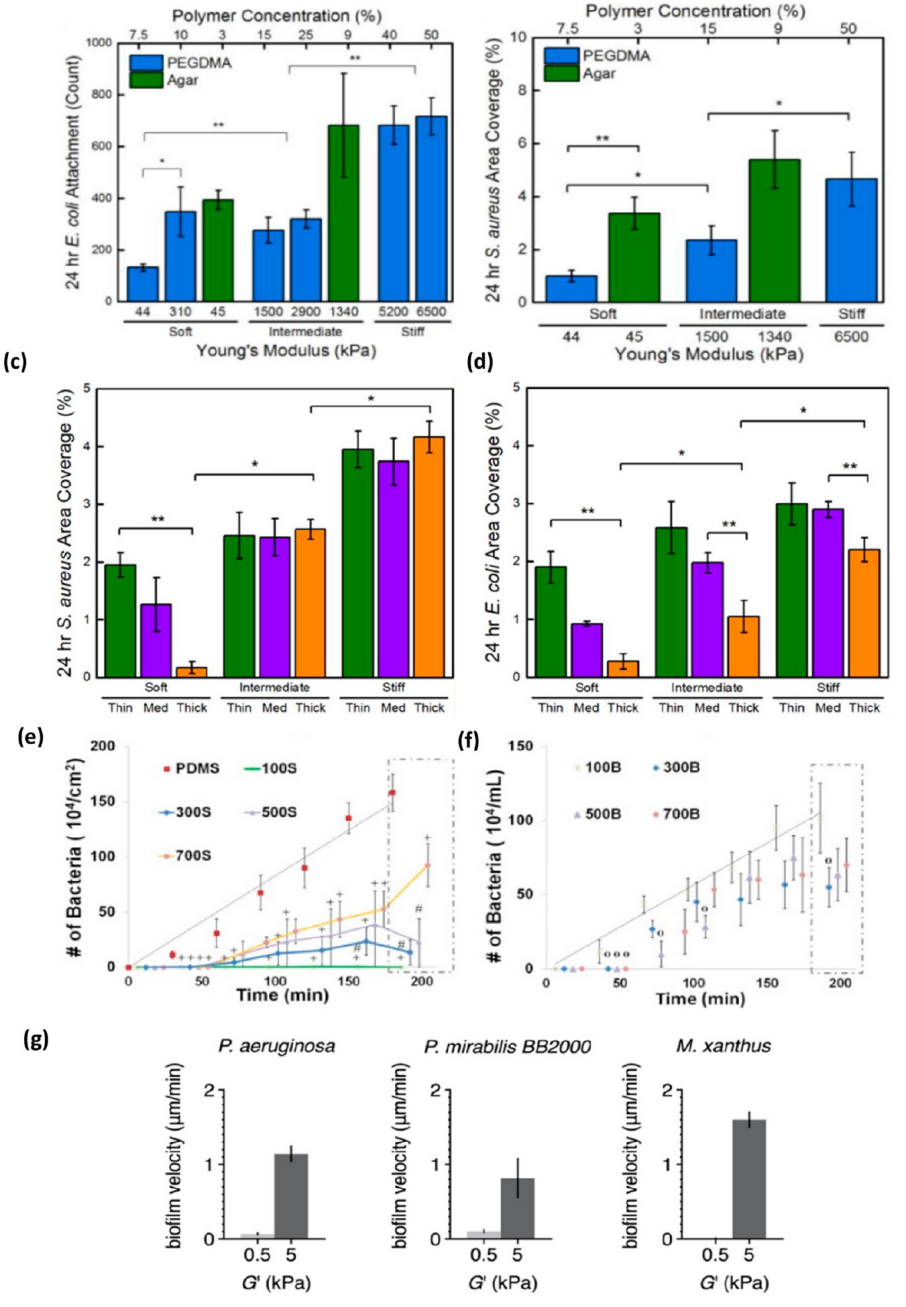
Studies indicating the effect of hydrogel stiffness and thickness on bacterial adhesion/growth. Increased growth of *E. coli* (a) and *S. aureus* (b) on agar and PEGDMA hydrogels with increased stiffness. Stiff agar hydrogels were not included as agar solubility limited its preparation. (a, b) Reprinted from ref (69). Copyright 2015 American Chemical Society. Increased growth of S. *aureus* (c) and *E. coli* (d) on thick poly(ethylene glycol) hydrogels with increased stiffness. (c, d) Reprinted from ref (65). Copyright 2018 American Chemical Society. Increased adhesion of *S. aureus* on the surface of ultrasoft PAAm hydrogels (500S and 700S) (e) and increased colonization of *S. aureus* in bulk of PAAm hydrogels (500B and 700B) (f). Increased growth of *P. aeruginosa*, *P. mirabilis*, and *M. xanthus* on PAAm hydrogels with increased stiffness (g). (e–g) Reprinted with permission from ref (72). Copyright 2016 Elsevier.

Hydrogel stiffness is the most widely studied property, yet there is no consensus on the correlation between hydrogel stiffness and bacterial adhesion and growth. Hydrogels are sometimes classified by their mechanical properties. In this section, we first highlight the studies on (1) conventional (relatively hard) hydrogels that seem to support the hypothesis of growth related to the stiffness and then (2) ultrasoft hydrogels where mixed results have been reported. Kolewe et al. demonstrated increased adhesion of motile bacteria *E. coli* and nonmotile bacteria *S. aureus*, when the stiffness of poly(ethylene glycol dimethacrylate) (PEGDMA) and agar hydrogels increased from 44 to 6500 kPa^69^ as shown in Figure 3a,b. They also demonstrated the influence of PEG hydrogel thickness with respect to hydrogel stiffness on bacterial adhesion.^65^ It was found that bacterial adhesion on thicker hydrogels increased with increasing stiffness (Figure 3c,d). While these studies suggest a positive correlation between bacterial growth and increased hydrogel stiffness and thickness, other studies present opposing results. For instance, when the stiffness of ultrasoft PAAm hydrogels was increased (PAAm-100, 654 ± 58 Pa; PAAm-300, 164 ± 33 Pa), a decrease in adhesion of *S. aureus* was observed compared to less stiff hydrogels (PAAm-500, *G*′ = 72 ± 16 Pa; PAAm-700, *G*′ = 17 ± 5 Pa).^72^ This study indicated that hydrogels with lower cross-linking densities (PAAm-500 and PAAm-700) showed improved *S. aureus* adhesion on the hydrogel surface (Figure 3e) as well as in the hydrogel bulk (Figure 3f) compared to highly cross-linked PAAm hydrogels. The authors suggest that the differences in hydrogel stiffness and bacterial growth are due to the inherent polymer characteristics, in particular the higher viscoelasticity of PAAm hydrogels compared to harder poly(ethylene glycol) hydrogels. They highlight the fact that stiff hydrogels provide a distinct 2D solid hydrogel–liquid bacterial suspension interface, where bacterial adhesion is reversible in nature. Conversely, in ultrasoft hydrogels a dynamic interaction between bacteria and hydrogel is enabled due to the 3D microenvironment of the hydrogel which promotes bacterial penetration in its porous network. Therefore, the study indicates that the hydrogel ultrasoftness is a desired characteristic for stable encapsulation of live bacteria. However, a recent study on identical PAAm ultrasoft hydrogels contradicts these findings, showing that bacterial growth and biofilm formation increased on stiff PAAm (*G*′ = 5000 Pa) compared to soft PAAm hydrogels (*G*′ = 500 Pa)^71^ (Figure 3g). More studies will have to be performed on PAAm hydrogels to address these contradictory findings.

Another factor influencing bacterial adhesion and growth is the hydrogel porosity, which is closely related to the hydrogel stiffness. For instance, Yañez et al. demonstrated the correlation between varying pore sizes of poly(2-hydroxyethylmethacrylate) (HEMA) hydrogels and bacterial growth.^70^ It was found that *S. aureus* and *P. aeruginosa* grew on hydrogel surfaces having pore sizes greater than 3.67 and 5.56 μm, respectively. Since bacteria are able to penetrate the hydrogel network and grow, these hydrogels may potentially be important for applications involving encapsulation of live bacteria in probiotic delivery and live bacteria/bacteriophages for wound management.

Hydrogel water content is an additional parameter that has been investigated for its influence on bacterial adhesion. Typically, the bacterial cell surface is hydrophobic in nature due to the presence of a peptidoglycan layer, lipopolysaccharides, and secretion of extrapolymeric substances which are rich in lipids. This suggests that bacterial adhesion would be favored on hydrogel surfaces with less water content. Very early studies indicated decreased adhesion of *P. aeruginosa* on contact lens hydrogels enriched with water compared to hydrogels with less water content.^75,76^ However, recently, a study indicated increased adhesion of *P. aeruginosa* and *S. aureus* on high-water-content contact lens hydrogels made out of polymacon compared to nesofilcon A, nelfilcon A, and omafilcon A low-water-content hydrogels.^77^ Contact lenses are typically developed from HEMA-based hydrogels; however, a fundamental difference between the above-mentioned hydrogels is the cross-linker and initiator used which alters the physicochemical properties of the hydrogels, thus altering the water content. These studies indicate that there is no significant correlation between hydrogel water content and bacterial adhesion. However, due to inherent hydrophobic bacterial characteristics, development of hydrophilic hydrogels may enable bacterial repulsion thus, enabling bacterial antifouling.

In addition to the aforementioned factors, bacterial motility has also been evaluated for its role in bacterial adhesion on hydrogel surfaces. A study has shown that bacterial motility organelles such as flagella, pili, and fimbriae could modulate bacterial adhesion on hydrogels. Guégan et al. demonstrated that *Pseudoalteromonas* species D41, a highly motile bacterium, exhibited greater adhesion on agarose hydrogels with 110 kPa stiffness compared to soft agarose with stiffness of 6.6 kPa.^67^ However, they also observed that *Bacillus* species 4J6, a nonmotile bacterium, adhered more strongly to soft hydrogels. The adhesion of nonmotile bacteria on hydrogels could be due to gliding movement and Brownian motion since they lack flagellar organelles. A further study indicated that, on agar hydrogels, nonmotile *Staphylococcus* exhibits comet and dendritic-like colony morphology through a process called “spreading”.^83^ These findings indicate that flagellum-driven bacterial motility alone does not define its adhesion on hydrogel surfaces.

Bacterial adhesion on hydrogels is a complex phenomenon influenced by both hydrogel and bacterial properties as described above. Therefore, it is important to consider the aforementioned factors for any bacterial/antibacterial applications.

Finally, we note that the microenvironment offered by the hydrogel is an important factor of influence on bacterial growth and proliferation. Hydrogels can be functionalized with bacterial nutrient medium to maintain an optimal microenvironment for bacterial cell hydration. The presence of nutrients within a hydrogel facilitates bacterial growth and proliferation due to nutrient consumption. For instance, a study evaluated the influence of different bacterial nutrient media encapsulated within 1% agarose hydrogels on bacterial growth.^66^ In particular, they demonstrated that the presence of tryptone, a peptide supplement important for bacterial growth, within nutrient-rich media functionalized agarose hydrogels improves bacterial growth compared to nutrient-minimal media. However, they also reported that the presence of tryptone along with bacteria increased the stiffness of agarose hydrogels as determined by compression tests with stress relaxation at 0.5, 2, and 5% strain values. The increased stiffness may be due to the cross-linking of bacteria with polymer chains. A further study indicated that PAAm hydrogels functionalized with bacterial nutrient media can be used as a substrate for 3D bacterial cultures.^78^ The authors reported improved bacterial growth on these hydrogels in comparison with similarly functionalized agar hydrogels, which suggested that the differences in bacterial growth could be due to the differences in the diffusion rates of nutrient media from hydrogels as a result of differences in PAAm and agar hydrogel stiffness. Even though it is known that bacterial nutrients are necessary to enable healthy and continuous bacterial growth, which is essential for secretion of bacterial metabolites, biofilm formation, and host–pathogen interactions, their incorporation in hydrogels (discussed below) may alter the hydrogels’ physicochemical properties.

## 4 Encapsulation and Release of Bioactive Elements

This section introduces different encapsulation strategies for incorporating desired bioactive elements within the hydrogel and their release mechanisms. The aqueous nature of hydrogels provides a conducive environment for encapsulation (and/or release) of bioactive elements. The interconnected porous hydrogel network enables stable encapsulation of bioactive elements by maintaining its functions and improving its longevity.^24,84−86^ In the context of bacterial studies and antibacterial applications, some of the active elements include antibacterial agents (antibiotics, bacteriostatic and bactericidal agents), bacterial growth media and supplements, sensing compounds such as redox indicators, metabolic substrates, cell viability dyes, and enzymes that perform their intended functions once released from the network. The hydrogel network acts as a molecular sieve by facilitating the selective transport of molecules with a particular size threshold from the hydrogel microenvironment. The transported molecules interact with the encapsulated bioactive elements, which enables their diffusion.^87^

### 4.1 Encapsulation

Typically, bioactive elements can be introduced within a hydrogel network (i) by post hydrogel synthesis, (ii) during hydrogel synthesis, or (iii) by embedded carrier systems. In (i), readily synthesized hydrogels are suspended in the bioactive element solution. The uptake of the bioactive elements into the hydrogel network is done via swelling. In (ii), the bioactive elements are mixed with hydrogel precursor solution for their incorporation within the hydrogel. For embedded carrier systems (case iii), bioactive elements are enclosed within carriers which are then embedded within the hydrogel network either by swelling post hydrogel synthesis or by mixing during hydrogel synthesis. The mechanism of bioactive encapsulation for each case is detailed below.

#### 4.1.1 Encapsulation by Swelling

Swelling is an important characteristic of a hydrogel that allows for encapsulation (and subsequent release) of bioactive elements. It is a process in which the bioactive elements are put in contact with the hydrogel and are entrapped within the hydrogel network via swelling. Hydrogels may readily absorb bioactive elements from the solvent until an uptake equilibrium is attained. As a result, a measurable volumetric change and alterations in physicochemical/mechanical properties are observed in the hydrogel.^1,2,6,88^ Depending on the bacterial/antibacterial application, it is important to determine a desired hydrogel with optimal swelling characteristics to enable encapsulation (and/or release) of active elements while maintaining its mechanical and structural stability. Two important parameters, namely hydrogel pores and the degree of hydrogel cross-linking, play a critical role in determining the swelling capacity of a hydrogel which in turn influences its mechanical properties.^89−91^ Hydrogels with large pore sizes such as macroporous (20–200 μm)^92,93^ and superporous (>100 μm)^94,95^ hydrogels have higher swelling capacities and thus exhibit increased elasticity and flexibility due to the expansion of the intermolecular hydrogel network and decreased glass transition temperature (*T*_g_) compared to nonporous and microporous hydrogels (20–60 μm).^96^

Hydrogels typically contain an uneven distribution of pores within the network, resulting in a polydispersity which is also largely determined by the route of hydrogel synthesis.^90^ For instance, free radical polymerization can result in molecular closed loops and dangling ends.^97^ This has the consequence of forming uneven porous hydrogel networks which are unsuitable for applications involving constant antibiotic release; however, it can be addressed by different techniques such as particle leaching, freeze-drying, gas foaming, electrospinning, micropatterning, and micromolding techniques to maintain a uniform pore size distribution.^90^ While hydrogel pores and interconnectivity determine the rate of swelling, the mechanism of solvent uptake is influenced by the nature/composition of the hydrogel.^89,98^ Typically, nonionic hydrogels swell by diffusion, where the solution containing bioactive elements migrates into the porous hydrogel network. In this case, hydrogels are under the influence of mixing and convective thermodynamic forces that allow the encapsulation of active elements into the hydrogel network until an equilibrium is achieved. Convection, advection, and capillary forces largely drive the movement of active elements into the hydrogel networks that contain large pore sizes.^89,98^ Ionic hydrogels comprised of charged moieties experience additional ionic interactions which attract hydrophilic molecules and thus improve solvent uptake.^89,98^

Recent studies have indicated the use of 3D printing to achieve desirable pores that allow encapsulation of bioactive elements by capillary effects and attainment of a rapid equilibrium state.^99−101^ By providing a protective environment, hydrogels can prolong the shelf life of bioactive elements such as enzymes to protect their catalytic functions, which is discussed in more detail in section 5. However, it is also noted that encapsulation of these molecules by swelling may require a long time, which may cause the inactivation of bioactive elements. This highlights the necessity to tune the cross-linking density and pore size for optimal swelling. As an alternative, mixing and hydrogel embedded carrier methods of encapsulation (discussed below) may be preferable.

Finally, we note that the ability of certain hydrogels to swell in response to microenvironmental changes such as changes in pH, temperature, chemical triggers, light, pressure, and electric and magnetic fields may also be exploited for bacterial sensing applications (described in section 5) and in the development of multifunctional composite hydrogels (described in section 6).

#### 4.1.2 Encapsulation by Mixing

Encapsulation by mixing during hydrogel synthesis is more suited for hydrogels that are microporous or nonswelling in nature due to their lower swelling capacities. Typically, soluble bioactive elements are mixed with the hydrogel pregel formulation, followed by hydrogel gelation which causes the encapsulation of bioactive elements within the hydrogel network. Depending on the route of hydrogel synthesis, bioactive elements may either be physically or chemically encapsulated within the hydrogel network. Hydrogels formed by physical interactions can encapsulate bioactive elements typically via hydrogen bonding, ionic and electrostatic interactions, stereocomplex formation, and DNA and protein interactions.^102,103^ Often, due to the relatively weak forces, physically cross-linked hydrogels are reversible in nature. A unique class of such hydrogels includes supramolecular hydrogels (described in detail in section 6.2.2) which exhibit excellent dynamic behavior due to high association and dissociation rates of physical forces.^104^ Chemically formed hydrogels via click chemistry reactions such as Schiff base mechanisms can either be reversible or nonreversible.^105−108^ In reversible reactions, the encapsulated bioactive elements are held together within the hydrogel network via dynamic covalent bonding. In contrast, the bioactive elements can be encapsulated via strictly nonreversible covalent bulk polymerization and free radical polymerization.

Having highlighted some important features of encapsulation by mixing, we highlight some of its limitations. The gelation of hydrogels in certain instances can be induced by an external catalyst such as light, temperature, or irradiation which may not be compatible with or may complicate the encapsulation of some bioactive elements. For example, in physically cross-linked hydrogels involving hydrogen bonding such as agarose, hydrogel formation is driven by temperature.^109^ This can be problematic as temperature-sensitive bioactive elements such as antibiotics, enzymes, and live bacterial cells rapidly deactivate when exposed to high temperatures. Thus, in this case, bioactive elements are introduced at the gelling temperature (∼45 °C) for encapsulation to prevent their inactivation. Hydrogels formed by chemical cross-linking such as light-dependent free radical polymerization require ultraviolet light as a trigger to initiate the polymerization. Typically, in such instances, certain bioactive elements such as bacterial cell growth indicators and nutrients rapidly precipitate due to deformation and aggregation of their respective structures/amino acids. Thus, in this case, bioactive elements should be encapsulated via swelling as described previously or by embedding hydrogel carriers as described in section 4.2. Furthermore, an important consideration while encapsulating and synthesizing hydrogels that depend on external catalysts is to ensure complete gelation. Indeed, several monomers/cross-linkers/initiators are known to be toxic and nonbiocompatible. It is therefore important to prolong the time of polymerization to ensure those are not released.

Encapsulation of bioactive elements within the hydrogel network by chemical cross-linking methods involving Schiff base reactions provide an alternative that limits the degradation of bioactive elements. In this case, gelation is facilitated by simple functional groups that are inherently found in the polymer or obtained by additional chemical cross-linker modification, thus avoiding the use of external catalysts, which are often toxic. In a Schiff base reaction, a reversible covalent imine bond is formed via the nucleophilic attack of an amine on the electrophilic carbon of aldehydes/ketones. An excellent feature of hydrogels developed by Schiff base reactions is their ability to self-heal due to the mild reaction conditions and reversibility enabling recovery of hydrogels after damage. In addition, the functional groups involved in Schiff base hydrogels are pH-sensitive and, thus, can potentially be utilized for bacterial sensing.

### 4.2 Release of Bioactive Elements and Hydrogel-Embedded Carriers

In some cases, it may be necessary to release bioactive elements. For example, in hydrogel-based bacterial detection platforms, the bacterial growth media and indicators encapsulated within the hydrogel are released when bacteria are introduced on the hydrogel. When a hydrogel is in contact with the subject material, the encapsulated bioactive elements are released from the hydrogel network into the subject microenvironment. The release typically occurs by diffusion, where the outward movement of bioactive elements from the hydrogel network is driven by the differences in the concentration gradient of bioactive elements between the hydrogel and its surrounding environment.

The release kinetics of bioactive elements is largely driven by the hydrogel pore size, which also plays a critical role in hydrogel swelling. Therefore, the strategy used for encapsulating bioactive elements within the hydrogel network determines the release behavior and effectiveness. Typically, a faster release rate of bioactive elements is observed in macroporous and superporous hydrogels due to the large pores when compared to nonporous and microporous hydrogels. This is not suitable for applications involving the release of antibacterial agents, especially in wound dressings and transdermal patches, as faster release can lead to localized acute toxicity.^98,102,110−113^ Moreover, the swelling mechanism typically results in a nonhomogeneous distribution of bioactive elements within the hydrogel network, whereas, in mixing, bioactive elements tend to uniformly distribute throughout the hydrogel network. Typically, it has been observed that hydrogels exhibit a rapid release of bioactive elements, where the initial rate of bioactive element release is directly proportional to the square root of time.^87^

In contrast, hydrogel-embedded carriers enable sustained release due to the encapsulation of bioactive elements within carrier systems which typically (i) display slower diffusion rates, (ii) improve the mechanical properties of hydrogels, and (iii) provide an additional protective barrier. The carriers are either dispersed or localized within the hydrogel network and provide an effective solution for release of bioactive elements over extended periods of time. In addition, the carriers are suitable for encapsulation of hydrophobic bioactive elements, whereas their encapsulation by swelling and mixing techniques exhibits poor encapsulation efficiencies. The carriers also stabilize the bioactive elements and provide a protective barrier, thus preventing them from degradation. A few examples of carriers used in the encapsulation of bioactive elements, mainly antibacterial agents, include (i) liposomes^114−117^ which are spherical vesicles containing at least one lipid bilayer formed from phospholipids, (ii) polymeric micelles^118−120^ which are colloidal particles formed due to the aggregation of surfactant phospholipid molecules in liquid, and (iii) carbon-based materials such as carbon nanotubes,^121,122^ carbon nanodots,^123,124^ cubosomes,^125,126^ and niosomes.^127^ The use of such carrier systems allows for high bioactive element loading capacity. For instance, in liposomes, due to the presence of distinct polar and nonpolar zones, hydrophilic and/or hydrophobic antibiotics can be encapsulated. Typically, the surface of carrier systems is charged or can be engineered to acquire surface charges which can be exploited for targeted antibiotic delivery. Furthermore, certain carriers with hydrophobic layers such as liposomes possess the ability to fuse with bacterial cells which can be potentially utilized for bacterial killing applications.^128,129^

Apart from conventional mechanisms, the release of active elements can be facilitated by external factors such as chemicals and environmental stimuli (see section 6). This type of triggered release broadens the application of hydrogels for bacterial sensing and development of multifunctional hydrogel platforms.

## 5 Hydrogels as Sensors for Bacterial Detection

Hydrogels are versatile materials that can inherently perform a wide range of functions and can be manipulated physically, chemically, and spatiotemporally to introduce desired bioactive elements as explained above. The sensing ability of hydrogels is another appealing characteristic making them a good choice for bacterial detection which is promoted by hydrogel properties such as porosity, swelling, permeability, and the ability of bacteria to interact with hydrogel surfaces as described in section 3. Depending on their sensing mechanisms, hydrogel-based bacterial sensors can be divided into three types: active sensors, functionalized sensors, and interfaced sensors. In active sensors, hydrogels are capable of sensing surrounding bacteria due to their inherent characteristics. Typically, external stimuli such as changes in pH, temperature, and secretion of metabolic enzymes and proteins, as a result of bacterial growth, transfigure hydrogel microproperties including pore size, degree of cross-linking, and ionic composition to produce significant macrostructural changes such as hydrogel swelling or deformation. In functionalized hydrogels, bacterial sensing is enabled by functionalizing the hydrogel surface/network with bacterial sensing elements that may also include BCEs for bacterial capture as detailed in sections 2.1 and 4.1. The third type, interfaced sensors, makes use of hydrogels at the interface of independent sensors such as optical, mechanical, and electrical external sensors to produce robust bacterial sensing platforms. This section highlights some of the intriguing features of active and passive hydrogels and their bacterial sensing mechanisms. Furthermore, we focus on surface/network functionalization aspects of hydrogels to enable bacterial sensing. Finally, bacterial sensing hydrogels are an integral part of multifunctional composite hydrogels that have been designed to specifically “capture and sense” various bacteria and “sense and treat” bacterial infections as discussed in section 6.

### 5.1 Active Hydrogels

#### 5.1.1 pH-Responsive Hydrogels

A feature of active hydrogels is their ability to undergo physicochemical and structural alterations by sensing external changes. An important class of active hydrogels that has dominated the field of hydrogel-based bacterial sensors is pH-responsive hydrogels,^130−135^ due to their ability to swell in the bacterial niche. Most bacteria reside and proliferate at neutral pH, while some can also thrive under acidic and basic pH conditions. During bacterial infections, it is observed that bacterial biofilms along with secreted proteins and environmental factors increase or decrease the pH of a system.^136−140^ For instance, in local burn wound infections, *S. aureus* and *Staphylococcus epidermidis* (*S. epidermidis*) are known to persist at a basic pH.^136^ In implants, bacterial contamination decreases the pH and creates an acidic environment.^141^ Therefore, pH-responsive hydrogels have great potential in bacterial sensing for detection of pathogenic bacteria and management of bacterial infections.

The underlying mechanism of the pH-dependent swelling/shrinking behavior of hydrogels is dependent on the concentration and availability of weak acidic and basic functional groups within the hydrogel.^142−145^ Typically, pH-responsive hydrogels are developed using monomers/polymers that contain acidic groups such as carboxylic and sulfonic acids and basic groups such as amines—primary, secondary, and ammonium salts which are ionized in basic and acidic microenvironments, respectively. Ionization of these functional groups is dependent on the acid dissociation constant (p*K*_a_) of the hydrogel polymer along with the surrounding pH.^142−145^ In a basic microenvironment, the pH is higher than the p*K*_a_ of the hydrogel polymer, which causes deprotonation of its basic functional groups. Therefore, the hydrogel carries a negative charge, which alters its osmotic pressure and attracts hydrophilic molecules resulting in hydrogel swelling. In addition, due to the high concentration of negatively charged groups, the polymer backbone of the hydrogel experiences electrostatic repulsion which further aids swelling.^145^ Conversely, in an acidic microenvironment, due to protonation of the hydrogel polymer, the hydrogel tends to shrink.^145^ Therefore, the swelling/shrinking nature of hydrogels is reversible and, in these cases, can be controlled by the microenvironmental pH. pH-responsive hydrogels have been developed in the context of multifunctional hydrogels, in which hydrogels are able to sense the bacteria and perform a wide range of downstream functions that are extensively addressed in section 6. pH-responsive hydrogels have been integrated within microfluidic platforms,^146^ electrochemical sensors,^147^ and fluorescent systems^148^ to improve their bacterial sensing performances. For instance, Tang et al. fabricated a microfluidic pH sensor from a pH-responsive chitosan hydrogel and poly(dimethylsiloxane) (PDMS) coated onto electrochemically etched porous silicon chips.^146^ They used it to evaluate the antibacterial susceptibility of bacteria in a solution containing different antibiotics via hydrogel swelling by Fourier transform reflectometric interference spectroscopy (FT-RIFS). The sensor enabled rapid bacterial confinement and detection within the microfluidic channels and, thus, reduced the time of antibiotic susceptibility testing. The group demonstrated *E. coli* detection by spiking different antibiotics in bacterial culture and introducing them in the microfluidic–electrochemical chip and evaluating antibiotic susceptibility within 2 h. Another example includes the fabrication of poly(vinyl alcohol)/poly(acrylic acid) integrated with a nanofiber light addressable potentiometric sensor that demonstrated *E. coli* detection in less than an hour with bacterial copies as low as 100 CFU/mL.^147^

#### 5.1.2 Thermoresponsive Hydrogels

Thermoresponsive hydrogels are an interesting category of active hydrogels that have been developed for bacterial sensing applications due to a characteristic reversible property known as volume-phase transition,^149−151^ which influences hydrogel swelling and shrinking behaviors. Typically, these hydrogels have been exploited to improve the efficiency of bacterial sensing through integration with external sensing devices or functionalization with bacterial sensing elements.^152−154^ Thermoresponsive hydrogels have been developed using amphiphilic polymers with hydrophobic and hydrophilic moieties held together via weak intra- and intermolecular hydrogen bonds whose association and dissociation in the presence of solvent molecules driven by a critical temperature are solely responsible for the dynamic “sol–gel” and “gel–sol” transitions.^155,156^ On the basis of the threshold value of critical temperature, thermoresponsive hydrogels are classified into two types: upper critical solution temperature (UCST) and lower critical solution temperature (LCST). When the transition of a polymer solution occurs from a solution state to the gelation state above a critical temperature, the hydrogels are termed LCST materials. At lower temperatures, the hydrophilic groups within the cross-linked hydrogels interact with the solvent water molecules via hydrogen bonding and are in a swollen state. As the temperature increases above the critical value, these hydrogen bonds are broken, resulting in shrinking by formation of coiled globular aggregates. In contrast, hydrogels formed by the transition of a polymer solution from a solution state to the gelation state below a critical temperature are known as UCST materials. Typically, LCST-based thermoresponsive hydrogels have been developed for bacterial sensing due to the gelling properties of polymers at temperatures close to ∼37 °C, which is an optimal temperature for bacterial growth. For instance, pNIPAAM, a most extensively studied hydrogel for various biomedical applications, due to its gelling property at 32 °C has been developed for bacterial sensing by integrating with a gold electrode conjugated electrically receptive graphene–nanoplatelet membrane.^153^ This study demonstrated the bacterial capture, growth, and sensing ability of pNIPAAM modified thermoresponsive hydrogels. Due to their gelling characteristics at physiological conditions, bacterial growth is favored, which causes the pNIPAAM polymer to shrink and alter the electrical properties of the integrated Au–graphene–nanoplatelets.

### 5.2 Bacterial Sensing Element-Functionalized Hydrogels

While some hydrogels are inherently active, others can be functionalized with bacterial sensing elements to equip bacterial sensing characteristics typically for hydrogels that exhibit important/desired features such as improved structural and mechanical properties, higher antibiotic loading capacity, enhanced bacterial adhesion, etc. which may not be offered by certain active hydrogels. Furthermore, active hydrogels can be functionalized with bacterial sensing elements to augment their sensitivity, specificity, and overall bacterial sensing performance.

#### 5.2.1 Enzyme-Mediated Bacterial Sensing by Hydrogels

**Figure 4:**
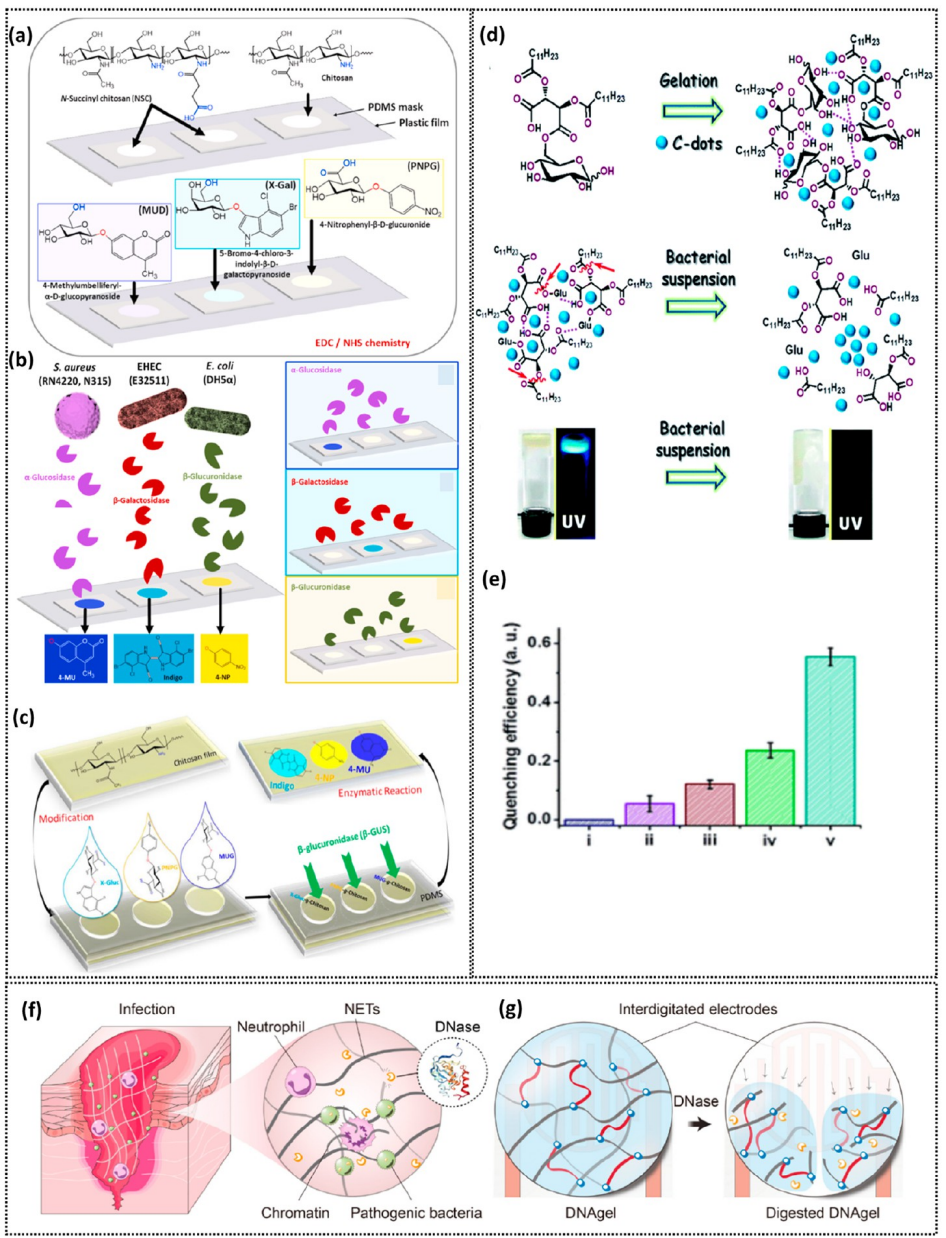
Bacterial sensing hydrogel platforms. (a) Arrangement of chitosan-PDMS plastic films and functionalization of chitosan hydrogels with metabolic substrates to enable enzyme-mediated bacterial detection. (b) Detection of specific bacteria via their unique secreted enzymes that convert the metabolic substrates within chitosan hydrogels to produce end products with distinct colors. (a, b) From ref (163). CC BY-NC-ND 4.0. (c) Schematic representing the fabrication of functionalized chitosan hydrogel film for enzyme-mediated *E. coli* detection. Reprinted with permission from ref (161). Copyright 2018 Wiley. (d) Schematic representing the formation of carbon dot hydrogel, collapse of hydrogel network due to bacterial esterases, and fluorescence transformation of hydrogels before and after bacterial treatment. (e) Degrees of fluorescence quenching: i, hydrogel without bacteria; ii, *P. aeruginosa*; iii, *B. subtilis*; iv, *S. aureus*; v, *B. cereus*. (d, e) From ref (164). CC BY 3.0. (f) Schematic representing the DNase secretion by the host during wound infection and (g) sensing mechanism of DNA gel which is degraded upon exposure to DNases causing alterations in capacitance of the sensor. (f, g) Reprinted with permission from ref (165). Copyright 2021 American Association for the Advancement of Science.

Bacteria are known to secrete characteristic enzymes during their growth and host infection process which can be utilized as a marker for investigating bacterial infections. For example, *P. aeruginosa* secretes an elastase enzyme, which is a virulence factor involved in host pathogenesis by hydrolyzing elastin in the host connective tissues.^157,158^ α-Glucosidase, a unique *S. aureus* identifier, hydrolyzes nonreducing (1→4)-linked α-d-glucose to produce α-d-glucose.^159^ Moreover, *S. aureus* can be differentiated from other staphylococcal species and *E. coli* strains due to the absence of β-galactosidase and β-glucuronidase activity.^160^ Therefore, hydrogels have been functionalized to recognize these unique enzymes for identification and characterization of pathogenic bacteria. Typically, enzyme recognition by hydrogels is facilitated by immobilizing reporter molecule tagged enzyme substrates on hydrogel surfaces.^161−163^ When these functionalized hydrogels facilitate bacterial adhesion and proliferation, bacterial enzymes access the substrates on the hydrogel and catalyze a hydrolysis reaction to produce chemical compounds emitting detectable signals. For instance, Jia et al. demonstrated multiplex specific detection of *S. aureus* strains, enterohemorrhagic *E. coli*, and *E. coli* Dh5a by immobilizing reporter tagged 4-methylumbelliferyl-α-d-glucopyranoside (target enzyme α-glucosidase) (MUD), 5-bromo-4-chloro-4-indolyl-β-d-galactopyranoside (X-Gal) (target enzyme β-galactosidase), and 4-nitrophenyl-β-d-glucuronide (PNPG) (target enzyme β-glucuronidase) substrates, respectively, on *N*-succinyl chitosan and chitosan hydrogel films via EDC-NHS chemistry^163^ (Figure 4a). As bacteria interact with the hydrogel, their characteristic enzymes hydrolyze the substrate to produce colorimetric chemical compounds. Using this strategy, the group demonstrated detection of *S. aureus* due to conversion of MUD to 4-methylumbelliferone (MU) by α-glucosidase generating a blue signal. Similarly, they were able to detect enterohemorrhagic *E. coli* due to the conversion of X-Gal by β-galactosidase producing indigo and *E. coli* Dh5a due to the conversion of PNPG to a yellow 4-nitrophenol (Figure 4b). Therefore, a distinct and independent differentiation of bacterial species and strain is observed in the hydrogel due to the unique colorimetric signals as a result of specific substrate hydrolysis by respective bacterial enzymes. Similarly, the group demonstrated multiplexed detection of *E. coli* strains via their secretion of β-glucuronidase, by patterning chitosan hydrogels with different metabolic substrates (Figure 4c).^161^ Alternatively, the direct chemical interaction between the secreted bacterial enzymes and hydrogels has been explored for detecting bacterial species.^164^ Typically, bacterial enzymes can induce the collapse of the hydrogel network by cleaving inter- and intramolecular bonds. For instance, Bhattacharya et al. demonstrated the ability of carbon dot hydrogels to distinguish *P. aeruginosa*, *Bacillus subtilis* (*B. subtilis*), *Bacillus cereus* (*B. cereus*), and *S. aureus* species based on the extent of chemical reactions between their secreted enzymes, namely esterases and lipases with the hydrogel. The carbon dot hydrogel was developed from 6-*O*-(*O*-*O*′-dilauroyltartaryl)-d-glucose cross-linked via ester bonds and is fluorescent in nature (Figure 4d). In the presence of bacteria, the secreted enzymes catalyze the hydrolysis of the ester bonds causing hydrogel deformation and subsequent fluorescence quenching (Figure 4d). The extent of hydrogel deformation and fluorescence quenching is unique to each bacterium (Figure 4e), thus enabling bacterial identification and differentiation.^164^ Xiong et al. developed a DNA hydrogel based wearable sensor from the covalent cross-linking of DNA strands with poly(ethylene glycol) diglycidyl ether equipped with DNase sensing characteristics. In wound infections, unlike commensal bacteria of the skin, pathogenic bacteria secrete a considerable number of DNases^165^ (Figure 4f). The enzymes cleave DNA molecules, which is essential for biofilm formation and bacterial evasion. Therefore, the DNA hydrogel undergoes deformation in the presence of pathogenic bacteria. Furthermore, the assay enables real-time wound infection management due to integration of the DNA hydrogels with interdigitated electrodes (Figure 4g) that detect hydrogel structural changes due to DNase activity followed by data analysis using near-field communication found on most smartphones.^164^ The opportunities resulting from the integration of hydrogels with external sensors for bacterial sensing applications are described in section 5.2.2.

#### 5.2.2 Hydrogels Interfaced with External Sensors

Typically, biological signal transduction in external sensors occurs via optical techniques, mass-based detection, and electrochemical methods involving amperometry, potentiometry, conductometry, and impedimetry.^166−168^ Active hydrogels have been integrated with external sensors to improve their bacterial sensing performances; see sections 5.1.1 and 5.1.2. In addition, hydrogels functionalized with bacterial sensing elements can also be integrated with external sensors. Such hydrogel interfaced sensors improve the sensing efficiency by signal amplification to produce measurable signals and produce simple and cost-effective bacterial detection platforms due to affordable external sensors. They utilize nontoxic and biocompatible hydrogels interfaced to a wearable sensor for rapid, real-time, and continuous monitoring of analytes for the management of bacterial infections.

Bacterial capture is a preliminary step in certain platforms involving hydrogel-interfaced external sensors for enabling the detection and enrichment of selective bacterial species. As discussed in section 2, BCEs can be immobilized on hydrogels for highly specific and sensitive recognition of specific bacteria. As an example, an antibody-immobilized porous silicon modified PAAm hydrogel interfaced with an optical sensor was developed for direct bacterial capture and detection; as low as 10^3^ CFU/mL within a few minutes was achieved. The oxidized porous silicon acts as an optical transducer element whose optical interference pattern is altered upon bacterial capture by the functionalized antibody–hydrogel biosensor platform.^35^ A few other examples of such sensing platforms can be found in articles by Khan et al. and Makhsin et al., respectively.^153,169^

## 6 Multifunctional Composite Hydrogels

Hydrogels can fulfill a range of functions in the context of bacterial and antibacterial studies as seen above. Only on rare occasions can individual hydrogels support all the desired properties. Therefore, composite scaffolds are put together to execute multiple functions. Such composite hydrogels are equipped with multiple bioactive elements within a single hydrogel network, or multiple functionalized hydrogels are combined for a synergistic performance. Therefore, a combination of appropriate hydrogels with desired properties and functionalization paves the way for the development of robust and intelligent hydrogel-based platforms.

In this context, the development of smart multifunctional composite hydrogels has mainly focused on antibacterial applications to specifically detect and eliminate pathogenic bacteria. Such hydrogels are designed for both diagnostic and therapeutic bacterial infectious disease management purposes. Their development is described below.

### 6.1 Multifunctional Bacterial Detection and Treatment Hydrogels

#### 6.1.1 “Capture and Respond” Hydrogels

**Figure 5:**
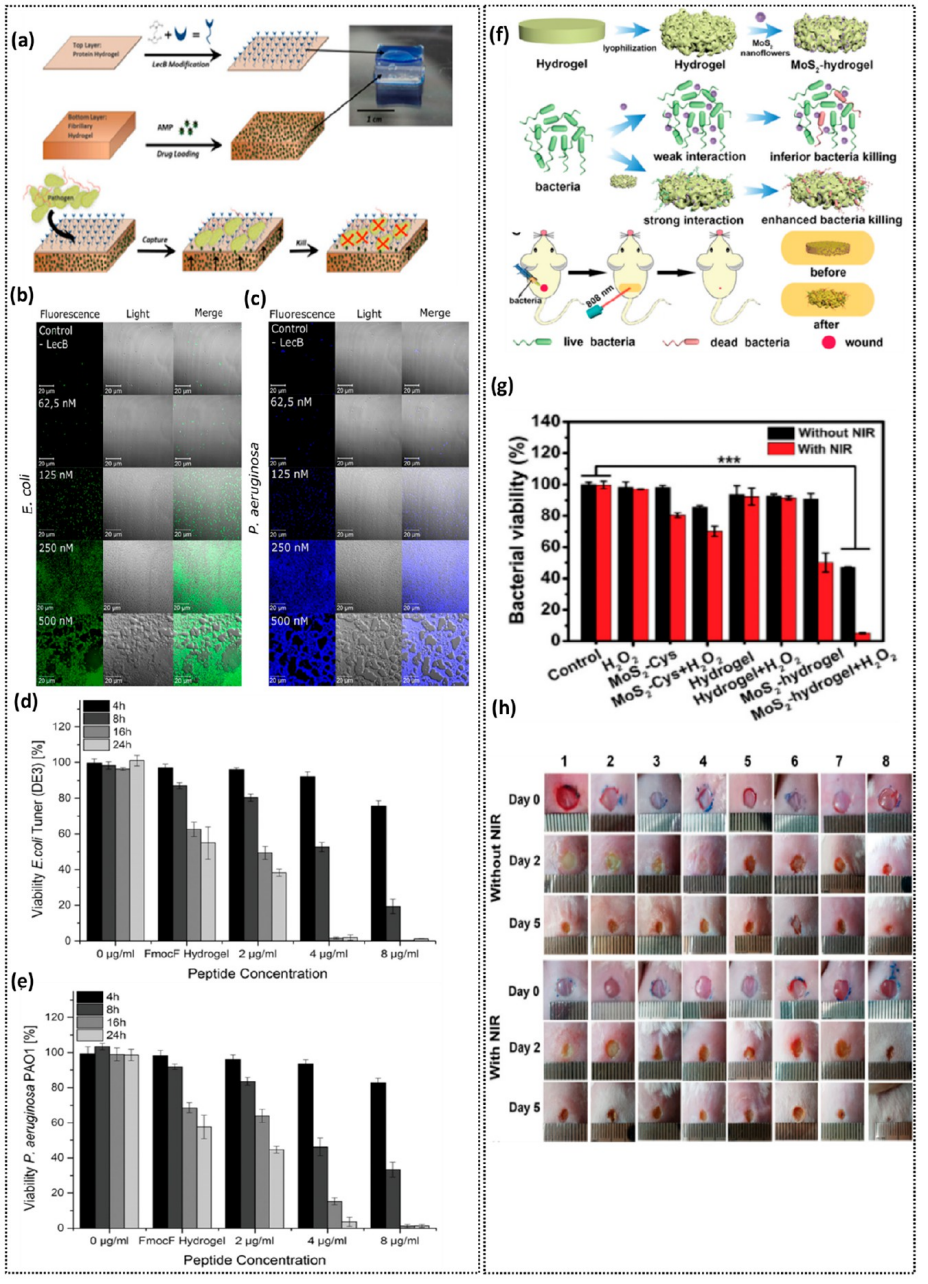
Multifunctional bacterial “capture and respond” hydrogels. (a) Schematic illustrating strategic “capture and kill” multifunctional hydrogel platform. The top layer constitutes serum albumin based hydrogel modified with BMOE cross-linker to immobilize lectin B for enabling bacterial capture. The bottom layer constitutes a fibrillary hydrogel encapsulated with AMPs for their release during bacterial contact resulting in bacterial killing. Increased capture of *E. coli* (b) and *P. aeruginosa* (c) with increasing concentration of lectin B. Effect of time and concentration of AMP encapsulated within hydrogels: after 24 h no *E. coli* cells (d) and *P. aeruginosa* cells (e) were viable. (a–e) From ref (25). CC BY 4.0. (f) Schematic representing functionalization of hydrogel with MoS_2_, antibacterial activity, and treatment of wound infections. (g) *E. coli* cell viability after treatment with and without MoS_2_ functionalized hydrogels in the presence and absence of NIR. (h) Evaluation of wound disinfection and healing upon treatment with MoS_2_ functionalized hydrogels with and without NIR. (f–h) Reprinted with permission from ref (33). Copyright 2019 Wiley.

Hydrogels functionalized with BCEs are typically composite hydrogels developed for rapid bacterial identification with subsequent downstream functions such as (i) antibacterial activity either by releasing the encapsulated antibacterial agent or due to the inherent antibacterial nature of the hydrogel or (ii) bacterial sensing for identifying the pathogen of interest. Such “capture and respond” hydrogels expand the utility of hydrogels for diagnosing and treating bacterial infections by selectively enriching bacteria from the biofluid/site of infection and performing downstream analysis. For instance, hydrogels functionalized individually can be structurally organized to produce a composite scaffold, where each hydrogel performs a unique function as demonstrated by Bodenberger et al.^25^ In this study, two functionalized hydrogels, (i) a serum albumin hydrogel functionalized with lectin B BCEs and (ii) a fibril-forming amino acid based hydrogel encapsulated with antimicrobial peptides (AMP), were structurally arranged to construct a composite hydrogel for selective capture and elimination of *P. aeruginosa*. Hydrogels functionalized with lectin B constituted the top layer, which interacts with the site of bacterial infection to capture pathogenic bacteria, and the AMP-functionalized hydrogels were situated below the top layer to enable the release of AMP upon bacterial capture for bacterial killing (Figure 5a). The lectin functionalized and AMP encapsulated hydrogel demonstrated successful bacterial capture, wherein the capture of *E. coli* and *P. aeruginosa* increased with increasing lectin concentration (Figure 5b,c), and 8 μg/mL AMP concentration eliminated viable bacteria within 24 h (Figure 5d,e). This bacterial capture and subsequent killing hydrogel platform demonstrate the applicability of composite hydrogels in detecting specific bacteria and treating bacterial infections, including those caused by multidrug resistant bacteria. This bacterial “capture and kill” platform is a good example of multifunctional composite hydrogels made by two different functionalized hydrogel systems.

Alternatively, “capture and kill” multifunctional composite hydrogels can be fabricated by functionalizing an individual hydrogel with multiple bioactive elements.^170,171^ To further enhance their antibacterial efficiency, some hydrogel platforms were endowed with photothermal/photosensitive agents for the treatment of bacterial infections using photothermal/photodynamic therapy (PTT/PDT).^33,171^ Briefly, these antibacterial therapies rely on light intensity and illumination time to exert antibacterial effects. When exposed to light, multifunctional hydrogels containing a thermoresponsive polymer or photosensitizer convert the light energy into chemical energy, resulting in hyperthermia and production of reactive oxygen species (ROS) at the site of infection to kill the bacteria. As an example, a multifunctional hydrogel was fabricated from a thermoresponsive positively charged hydrogel: pNIPAAM-DMPA functionalized with MoS_2_ moieties and H_2_O_2_ by Sang et al.^33^ (Figure 5f). The positively charged hydrogel electrostatically confines bacteria to the surface of the hydrogel. Upon attachment, the MoS_2_ moieties induce antibacterial activity due to their inherent antibacterial nature. MoS_2_ is a photosensitizer; therefore, when it is exposed to NIR (near-infrared), MoS_2_ induces local hyperthermia/ROS production which is accelerated by H_2_O_2_, enabling rapid bacterial eradication (Figure 5f–h). As the hydrogel is thermosensitive, it can undergo physicochemical alterations due to increased temperature; however, the study demonstrated that the hydrogel did not interfere with the photothermal activity facilitated by MoS_2_. Further, the study demonstrated excellent tissue recovery by MoS_2_ + H_2_O_2_ hydrogels with NIR. Therefore, the study indicated the combinatorial effects of a single hydrogel with multifunctionalities for synergistic bacterial eradication and acceleration of the wound healing process.

As seen above, “capture and respond” multifunctional composite hydrogels enable the recognition of specific bacteria followed by their eradication. Thus, these hydrogels can potentially be utilized for various applications including hydrogel-based bacterial biosensors for determination and elimination of specific causative bacterial species, bacterial antifouling platforms to avoid bacterial attachment and growth, and localized/targeted antibacterial and wound healing therapy.

#### 6.1.2 “Sense and Treat” Hydrogels

**Figure 6:**
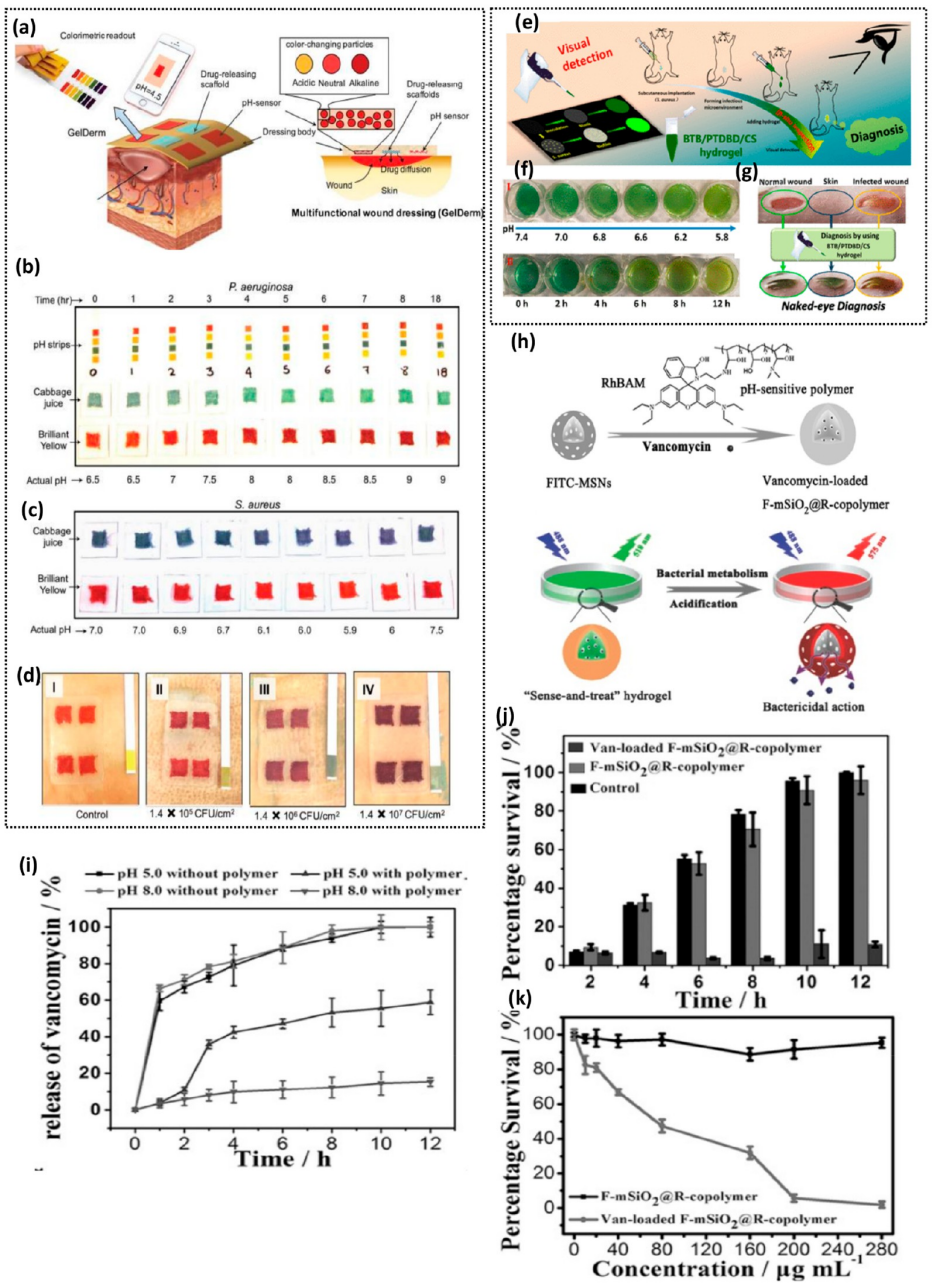
Multifunctional bacterial “sense and treat” hydrogels. (a) Schematic illustrating the application of multifunctional GelDerm wound dressing functionalized with pH-sensitive and drug-eluting compounds. pH variations for *P. aeruginosa* (b) and *S. aureus* (c) on hydrogels in comparison with commercial pH strips. (d) Colorimetric detection of bacterial infection in pig skins by multifunctional hydrogels indicating increased color intensity with increasing bacterial concentration. (a–d) Reprinted with permission from ref (135). Copyright 2017 Wiley. (e) Schematic illustration of multifunctional bromothymol blue (BTB)/near-infrared-absorbing conjugated polymer (PTDBD)/thermosensitive chitosan (CS) hydrogel for visual detection and diagnosis of bacterial infections. (f) pH-based colorimetric detection of bacterial growth via multifunctional BTB/PTDBD/CS hydrogel due to change in color from green BTB to yellow. (g) Images indicating multifunctional BTB/PTDBD/CS hydrogel mediated diagnosis of bacteria-infected wounds in mice. (e–g) Reprinted from ref (171). Copyright 2020 American Chemical Society. Schematic representing (h) the preparation of vancomycin-loaded “sense and treat” hydrogel and their mechanism of action for bacterial elimination. (i) pH- and polymer-dependent release kinetics of vancomycin from the “sense and treat” hydrogel. (j) Percentage survival of bacteria over time upon hydrogel treatment. (k) Percentage survival of bacteria over time upon treatment with hydrogels containing varying concentration of nanoparticles. (h–k) Reprinted with permission from ref (133). Copyright 2015 Wiley.

Significant research has focused on developing multifunctional composite hydrogels that can sense and subsequently treat bacterial infections using antibiotics. Typically, such hydrogels are fabricated by functionalizing bacterial detection molecules including molecules acting as BCEs and pH-sensitive dyes within the hydrogel network. In addition, the inherent bacterial sensing capabilities of the hydrogel via electrostatic interactions and alterations in their physicochemical properties have been exploited for bacterial sensing. A facile and efficient multifunctional alginate hydrogel (GelDerm) was developed with potent bacterial sensing and antibacterial characteristics for wound management via incorporation of different pH-sensitive dyes (cabbage juice and brilliant yellow)^135^ (Figure 6a). This designed hydrogel clearly distinguished *P. aeruginosa* and *S. aureus* within a broad pH range (Figure 6b,c). Further, the hydrogel was able to detect bacterial infections in pig skin, where the intensity of the encapsulated colorimetric dye increased with increasing bacterial concentration (Figure 6d). The hydrogel encapsulated an antibiotic, gentamicin sulfate, which was released upon sensing bacteria for their elimination. Further, the multifunctional hydrogel was introduced into Mepitel, a commercially available nonmedicated wound dressing and integrated with iDerm smartphone software for enabling its clinical utility by patients and healthcare professionals in a cost-effective way. Similarly, multifunctional agarose hydrogels were developed by encapsulating multiple bioactive elements to effectively sense and treat bacterial infection.^133^ Briefly, the hydrogel was developed by encapsulating two pH-sensitive moieties, namely fluorescein isothiocyanate (FITC) doped mesoporous silica nanoparticles (MSNs) containing a pH-sensitive polymer and a rhodamine derivative which was grafted on FITC-MSNs (Figure 6h). These pH-sensitive moieties are responsible for sensing the bacterial habitat and producing a fluorescent signal upon detection. The pH-sensitive polymer contained vancomycin, which was released subsequently. The study showed a pH-dependent release of vancomycin due to the pH-sensitive polymer compared to hydrogels without the polymer at different pH conditions (Figure 6i). Upon excitation at λ = 488 nm and in the bacterial microenvironment, the hydrogel displays a green to red color change due to pH shifts, thereby triggering the pH-sensitive polymer within the agarose hydrogel to release vancomycin and eradicate bacteria. This multifunctional hydrogel reduced *E. coli* viability within 12 h of exposure (Figure 6j) and demonstrated a decrease in *E. coli* survival with increasing concentrations of pH-sensitive nanoparticles (Figure 6k).

Furthermore, “sense and treat” hydrogels have increasingly been used in combination with PTT/PDT to treat bacterial infections. Thermoresponsive hydrogels present a great scope for the development of intelligent multifunctional composite hydrogels as mentioned briefly in section 6.1. Due to their temperature sensitivity, excited thermoresponsive hydrogels undergo structural/physicochemical alterations resulting in on-site bacterial eradication via release of encapsulated antibiotics, or ROS/hyperthermia. For instance, a strategic visual bacterial detection and on-site treatment of bacterial infections was enabled by the development of a multifunctional composite thermoresponsive chitosan hydrogel^171^ (Figure 6e). The hydrogel was encapsulated with bromothymol blue, a pH-sensitive dye which altered from green to yellow indicating *S. aureus* growth and its acidic environment (Figure 6f,g). Upon sensing, the thermoresponsive hydrogel underwent gelation at physiological temperature while simultaneously being exposed to near-infrared light which enabled the release of an encapsulated antibacterial agent, β-glycerophosphate, and induce localized treatment via hyperthermia. This study also highlights the application of thermoresponsive hydrogels as injectable hydrogels (section 6.2.1), where the raw materials essential for hydrogel formation along with multiple bioactive elements are introduced at the site of infection for targeted infection detection and diagnosis.

### 6.2 Multifunctional Antibacterial Therapeutic Hydrogels

#### 6.2.1 Injectable Hydrogels

Injectable hydrogels are increasingly preferred for therapeutic applications to enable site-directed delivery of therapeutic agents such as antibiotics for bacterial elimination and treatment of infections due to (i) their minimal invasiveness, (ii) their ability to take the form of irregular surfaces in situ/in vivo which enables maximum interaction between the hydrogel and the site of infection, and (iii) biodegradability due to which the hydrogels naturally dissolve in situ/in vivo over a particular timeframe, typically after a therapeutic window. It is important to note that injectable hydrogels are either physically or chemically cross-linked and are synthesized typically from biocompatible polymers which are either naturally occurring (chitosan, alginate, silk, gelatin, etc.) or synthetic (PEG, poly(lactic-*co*-glycolic acid), polylactic acid, etc). Essentially, these hydrogels possess in situ gelling characteristics at their site of introduction in liquid form. The “injectability” feature of such hydrogels is influenced mainly by microenvironmental parameters, typically physiological temperatures that induce hydrogel polymerization. Bioactive elements, especially antibiotics, are mixed with the hydrogel pregel solution and injected at the site of infection, which causes the hydrogel solution to polymerize due to temperature changes. Therefore, thermoresponsive hydrogels have been widely utilized as injectable forms for antibiotic delivery.^172−177^

Studies have also indicated the development of versatile multifunctional composite injectable hydrogels based on nonthermoresponsive hydrogels.^178−181^ For instance, an injectable functionalized composite hydrogel developed from coordinative cross-linking of silver nitrate with thiolated PEG was fabricated.^181^ It demonstrated excellent antibacterial activity against *S. aureus* due to silver ions embedded within the hydrogel and concurrently facilitated self-healing by repair of diabetic wounds due to the encapsulated angiogenic agent desferrioxamine.

#### 6.2.2 Supramolecular Hydrogels

The self-healing characteristic of supramolecular hydrogels, which are reversible physically cross-linked hydrogels with self-assembling properties, has been used for many antibacterial applications. The “self-healing” properties of these hydrogels are attributed to the dynamic reversibility due to the weak inter/intramolecular hydrogel network based on physical interactions such as hydrogen bonding, electrostatic interactions, ionic bonding, coordination complexes, and host–guest interactions. Typically, these interactions enable the self-assembly of hydrogel constituents resulting in their gelation.

Peptide-based hydrogels are important supramolecular hydrogels developed for antibacterial applications.^182−184^ Antimicrobial peptides, which are small peptide molecules exerting antibacterial effects, self-assemble due to their cationic and hydrophobic residues. A study by Wan et al. showed the self-assembly of nonionic peptide amphiphiles into hydrogels, where the lysine residues of the peptides exhibited pH responsiveness which determines its gelation and antibacterial activity.^185^ It was observed that, upon self-assembly, hydrogels without any lysine units exhibited poor antibacterial activity compared to hydrogels with lysine units (*n* = 1).

Supramolecular hydrogels can be integrated into multifunctional composite platforms, for example, injectable hydrogels to enhance antibacterial activity and promote accelerated tissue recovery. For instance, a supramolecular hydrogel based platform using chitosan grafted with β-cyclodextrin and adamantane was developed for wound healing applications and demonstrated excellent antibacterial activity and enhanced recovery of damaged wounds.^186^ The hydrogel was functionalized with graphene oxide, which along with the inherent antibacterial nature of chitosan and chitosan modified with quaternary ammonium inhibited bacterial proliferation.

## 7 Conclusion and Future Outlook

The continuous rise in bacterial infections and antimicrobial resistance requires a simple solution for the detection and identification of bacteria, their studies, or the treatment of bacterial infections. Hydrogels have proved to be very well-suited for these applications, due to their versatility and multifunctional nature. They offer a multitude of properties that can be exploited to develop smart multifunctional composite bacterial/antibacterial biomaterials. The development of such systems is a multidisciplinary endeavor that involves working at the interface of polymer chemistry, engineering, and biomedical sciences.

In this review we highlight the salient features that will enable the development of hydrogels for bacterial capture, adhesion, growth/antibacterial activity, and bacterial sensing. In particular we review chemical modifications, physicochemical and structural properties, and encapsulation/release of bioactive molecule strategies and how they can be combined to fulfill the functions highlighted above. This review highlights the emergence of multifunctional hydrogels by the combination of the aforementioned hydrogel features, functionalization strategies, and mode of action that have the potential to provide all-in-one solutions for bacterial infection management. The two types of multifunctional hydrogels, namely the composite hydrogels, comprised of a number of single function hydrogels, and the multifunction hydrogels made of a single hydrogel endowed with multiple functions, are reviewed. The synthesis and process limitations are reviewed in detail, and opportunities are highlighted.

The development and application of hydrogels for bacterial/antibacterial studies are promising and has advanced over the years. However, to a large extent it is limited to fundamental research. We anticipate that the development of multifunctional composite hydrogels will open new avenues for their translation into clinical settings or at the point of care for the management of bacterial infections and antimicrobial resistance. Furthermore, due to their ability to functionalize with one or more bioactive elements and their sequential performance, multifunctional composite hydrogels may act as a self-standing diagnostic and/or therapeutic platform. Importantly, such advanced hydrogels have the potential to make a significant difference in the field as they provide an interactive substrate which can be designed to recognize specific bacteria and thereby perform desired bacterial/antibacterial functions. In this context, the design, development, and performance of multifunctional composite hydrogels could be enhanced through an in-depth understanding of hydrogel properties and their functionalization to immobilize multiple bacterial capture agents, with encapsulation of multiple bioactive and sensing elements. Furthermore, the understanding of the cellular and molecular mechanisms of bacterial adhesion with respect to hydrogel structural and/or physicochemical properties could enable the advancement of efficient bacterial growth/antifouling hydrogel platforms and coatings. Therefore, multifunctional composite hydrogels have the potential to overcome traditional clinical challenges associated with bacterial infection, disease detection, and diagnosis. We note that the strategies highlighted in this review are relevant to other biomedical applications and can be adapted to different cell types.
